# Aging-friendly design strategies for community public service facilities based on context analysis

**DOI:** 10.3389/fpubh.2025.1649904

**Published:** 2025-11-12

**Authors:** Yang Li, Ruolin Zhao, Xu Zhang

**Affiliations:** School of Arts, Tianjin University of Technology, Tianjin, China

**Keywords:** aging-friendly design, older adults communities, situational needs, public service facilities, micro-transformation strategy

## Abstract

**Introduction:**

Against the backdrop of China’s continuously aging population, adapting public service facilities in older urban communities for the older adults has become an important issue in the fields of urban and rural planning and community governance. Most existing studies focus on static functional optimization, ignoring the dynamic needs of the older adults in different situations. This study aims to reveal the dynamic characteristics of the behavioral needs of the older adults in multiple dimensions such as time, space, and social interaction based on the context theory.

**Method:**

Through field investigation and questionnaire analysis, combined with the analytic hierarchy process, we quantify the demand weights of the older adults and semantic judgment methods were introduced as supplementary verification.

**Discussion:**

Based on this, we propose strategies such as behavior-oriented flexible facilities, time-responsive light environment regulation, “behavioral overlap area” design for space composite utilization, and data-driven lightweight iterative mechanisms. These provide approaches to aging-friendly renovation that take into account both accuracy and implementability for resource-constrained older communities, addressing the cognitive gap of situational dynamics in traditional designs.

## 1 Introduction

Accelerated aging means that the older adults now occupy a more important position in urban communities (1–6). As a product of early urbanization processes, older communities are places where a large number of older adults residents carry out their daily lives (7–9). However, both the infrastructures and public service facilities in these areas are also aging (10–15), and the specific needs of the older adults have not been fully considered (16), leading them to experience increasingly prominent difficulties and inconveniences in community life (17–20). To address this issue, aging-friendly renovations have become an important topic in current urban community construction practices (21, 22). Aging-friendly design for public service facilities in communities of older adults people not only concerns their basic needs, but also directly affects their physical and mental health and quality of life (23). Most of the existing aging-friendly designs focus on functional hardware renovations (22), mainly concentrating on optimizing facility functions and space renovations, such as improving road paving for the safety of older adults travel or optimizing the layout of recreational and fitness facilities. However, most of these studies remain at the static design level and lack systematic quantitative analysis of the behavioral patterns, spatio-temporal contexts, and individualized needs of the older adults. This study attempts to fill this gap through context theory and multi-criteria decision-making methods.

In communities of older people, spatial planning, infrastructure and public service configuration present many problems, directly affecting quality of life. First, limited and unevenly distributed public space (24) leads to a lack of sufficient activity venues and clear functional activity areas for older adults residents (25), meaning they have nowhere to carry out daily exercise or socialize (26). Second, aging infrastructures and a lack of aging-friendly design also affect safe travel (27). Public service facility delays have also exacerbated the problem of adaptation for the older adults. Existing fitness equipment, seats and other facilities have singular functions (28), meaning they struggle to meet the increasingly diverse needs of the older adults. Shortcomings in this regard are especially obvious in terms of social and leisure activities. In addition, the environmental quality of older communities is generally poor. Problems such as insufficient greening, severe noise pollution, and uneven ground have reduced activity willingness and quality of life for older adults people (29). Similar studies abroad also show that the activities of the older adults are greatly influenced by spatial environment, social facilities and daily behavioral habits, but most of them are based on questionnaires or observations, lacking quantitative methods based on situational analysis.

At present, adapting and renovating old communities for the older adults should not merely remain at the level of infrastructure repair and functional improvement. Instead, we must begin with actual usage needs, systematically analyzing common resident demands and individualized differences for public service facilities. Only by gaining an in-depth understanding of the specific demands of the older adults—in various aspects such as daily life, leisure and fitness, and social interaction (30–33)—can a more scientific and reasonable basis be provided for aging-friendly public service facility design for older communities. Compared with traditional research on older adults-friendly renovations, this study not only focuses on the optimization of facility functions but also emphasizes the interaction among the behavioral, temporal and spatial dimensions of the older adults. It quantifies the demand weights through multi-criteria decision-making methods to provide the community with scientific, operational and low-cost design solutions.

Based on this, this study focuses on the following questions: What are the core contextualized needs of the older adults in old communities? What are the priorities of these demands in terms of time, space and behavioral dimensions? What mismatches exist between the current elder kindness renovations and the demands? The research objective is to identify and quantify the needs of the older adults through the context theory framework and the multi-criteria decision-making method (AHP and MACBETH) system, and propose operational design strategies for aging-friendly public service facilities to achieve low-cost, context-responsive and sustainable community renewal paths.

This article is structured as follows: Section 2 presents a literature review of the contextual theory. Section 3 presents the methods of this study, semi-structured interviews and analytic hierarchy process, supplemented by Measuring Attractiveness by a Categorical Based Evaluation Technique. Section 4 analyzes the demands of older adults people regarding public service facilities in different situations. The results and quantitative analysis are presented in Section 5, which are then discussed in Section 6; strategies are also proposed here. Section 7 summarizes the significance of this study and puts forward suggestions for future research.

## 2 Theoretical review

### 2.1 Situational theory

Context represents the combination of subjective consciousness and objective existence within a certain period of time, including characters, time, surrounding environment, behavioral tasks, geographical location, etc. In current research, the most widely applied definition is that proposed by Dey et al. (34): a situation is any information about the characteristics of an entity, which is a person, place, or object related to the interaction process between the user and the application. Situation theory research began with Schicit et al.’s (35) proposal of situation awareness in 1994. That is, a system can actively perceive changes in a user’s situation through context-aware computing capabilities, providing timely and accurate information and services according to their current situational needs. With further research across various fields, the study of situational theory has gradually become systematic. Table 1 is a summary of definitions of context in relevant studies, beginning with the dimension of time.

**Table 1:** Definition of situation according to some scholars.

Scholar	Proposed time	Specific definition
B. Schilit	1994	Location, people and the surrounding environment, and their changes over time
Ryan, N	1997	Geographical location, identity, time, environment
Dey A. K	2000	All the information about the entity characteristics during the interaction process between the user and the system
J. Grudin	2003	Location, identity, status, object, group, etc.
Bardram	2009	The overall human activities, activity behaviors and process execution

### 2.2 Foundational theories

Safety is a basic requirement for community public service facilities (36). Given that mobility is limited and reaction speed slow down later in life, designs in this regard should pay special attention to safety (37). Adequate lighting facilities and convenient emergency call systems should be provided to create a safe and reliable living environment for the older adults. These design details can not only enhance safety for daily activities, but also strengthen trust and community belonging. The convenience of the facilities’ layout directly affects the usage experience for older adults residents (38). Public service facility distribution in older communities often fails to cover the main areas of activity. This distribution pattern leads to a low utilization rate, presenting difficulties in meeting the actual needs of the older adults. Furthermore, types of public service facilities in older communities are limited, lacking facilities tailored to the special needs of older adults people. Social interaction is also crucial for their mental health (39). Community environment construction should not only provide basic living places, but also create a multi-functional space integrating leisure, entertainment, fitness and social interaction (40); in this way, we can better meet the diverse outdoor living needs of such residents, promote interaction and communication among them, effectively alleviate their sense of loneliness (41, 42), and enhance their sense of community belonging.

## 3 Materials and methods

In the weighting process of the index system in this study, the analytic hierarchy process was employed. This method enables the structural decomposition of complex problems and determines relative weights through the construction of a judgment matrix. AHP is particularly well-suited for research in social sciences and spatial planning, where problems typically exhibit clear hierarchical structures and involve quantifiable expert knowledge. It effectively integrates qualitative judgment with quantitative analysis, thereby enhancing the systematicity and transparency of the weighting process (43–45). In this study, AHP not only quantifies the relative importance of various evaluation indicators, but also ensures the logical consistency of expert judgments through consistency checks. Consequently, it has been widely adopted in related research areas such as urban elder kindness environment assessment and optimization of facility layouts. Quantitative data were analyzed using SPSSAU.

The MACBETH method fundamentally relies on a qualitative description of the “differences in attractiveness” across various evaluation criteria (46–48). This is achieved by comparing two indicators through a semantically distinct approach, and subsequently translating fuzzy linguistic terms into numerical weights with the aid of a decision support system. As a result, the method enables both the quantification of qualitative judgments and the consistency verification of the judgment matrix (49, 50).

### 3.1 Research framework

See Figure 1.

**Figure 1:**
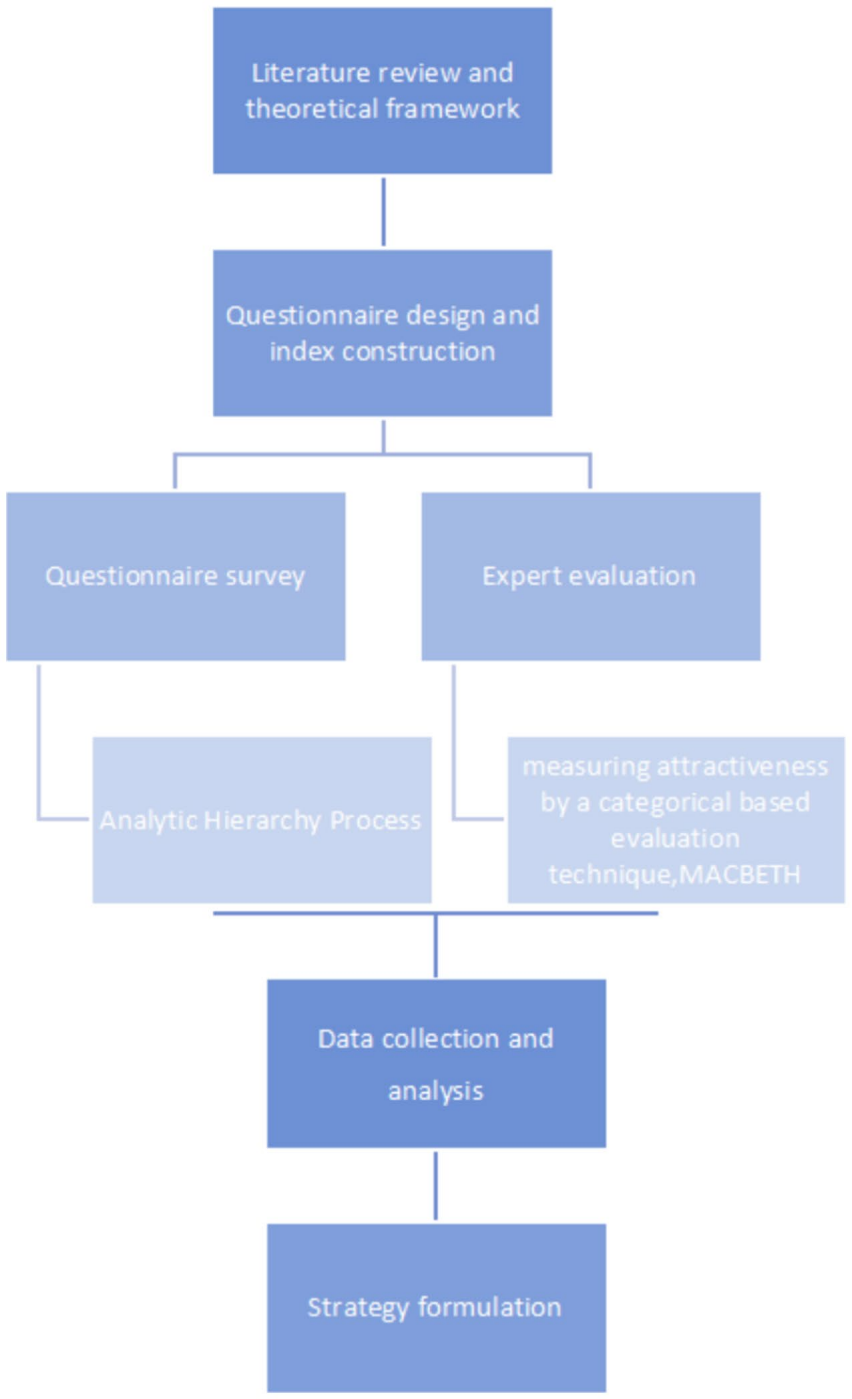
Flowchart.

### 3.2 Questionnaire survey

**Figure 2:**
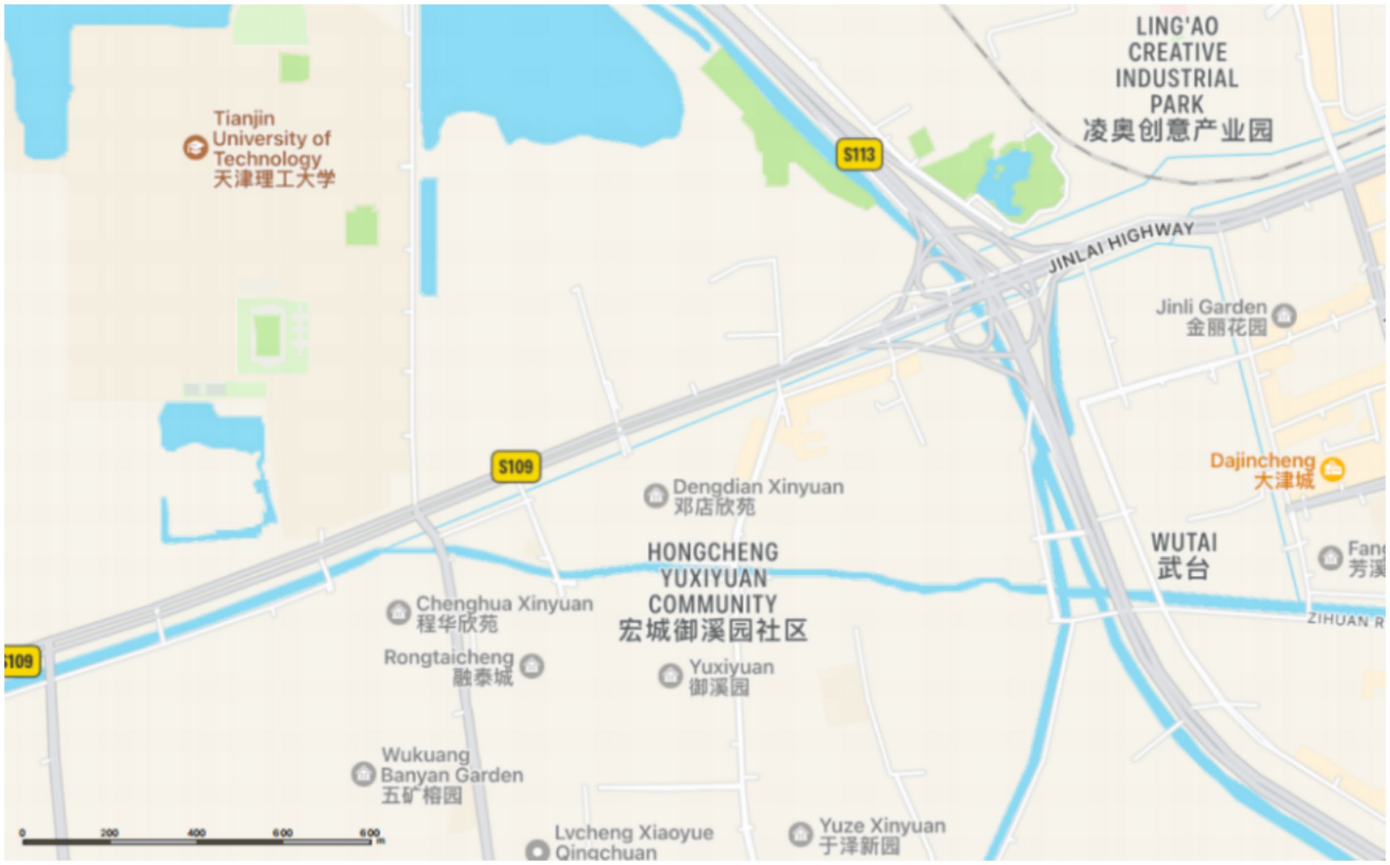
Geographical location map.

This study adopted the questionnaire survey method to collect feedback from residents and managers in Dengdian Xinyuan Community, Xiqing District, Tianjin, in order to analyze the differences in needs demonstrated by older adults people when using community public service facilities. Dengdian Xinyuan was completed in 2000 and has a residential area of 140,000 square meters. The community comprises a total of 1,550 households and is equipped with supporting facilities including a kindergarten, a health service center, a 7,000-square-meter vegetable market, and a 4,000-square-meter cultural square. Within a 3-kilometer radius, there are five universities. Commercial amenities also encompass building materials and home furnishing markets. Additionally, the area is well-served by multiple bus routes. The community’s geographical location is illustrated in the accompanying Figure 2.

It has the typical characteristics of most older communities. The community’s public space utilization rate is low, service facilities are growing old, and the population is aging severely. The research subjects of this questionnaire include three categories: older adults people who have lived in the community for more than 15 years, community managers and members of the residents’ committee. By collecting the evaluations and suggestions of different groups on the use of public service facilities, we ensured data representativeness and a broad multi-dimensional analysis.

### 3.3 Questionnaire content

The design of the questionnaire combines three types of information sources: literature review, field observation and expert interviews. Firstly, it drew on the existing relevant research results on urban elder kindness Spaces, behavioral geography of the older adults and community facility construction, providing theoretical support for the determination of the dimensions and contents of the questionnaire. Secondly, through the on-site investigation in the study area in the early stage, a series of practical problems in the use of facilities were identified, further supplementing and specifying the content of the questionnaire. Finally, four experts from the fields of urban planning, older adults care services and sociology were invited to review and revise the initial draft of the questionnaire to ensure its professionalism and practical relevance.

The interview questions in this study were developed based on context theory and existing research on the behavioral characteristics of the older adults within community settings. The primary objective is to systematically identify the public service facility needs of the older adults across varying temporal, spatial, and behavioral contexts. Specifically, the interview framework encompasses three key dimensions: Behavioral Dimension: This includes daily routines, leisure activities, physical exercise habits, and social interaction patterns of the older adults. Temporal and Spatial Dimension: This refers to activity preferences and facility usage patterns exhibited by the older adults across different times and within various community spaces. Facility Experience and Suggestions for Improvement: This dimension explores the older adults’ experiences with existing facilities, perceived inconveniences, and expectations for future enhancements.

Each set of interview questions aligns with the research objectives and serves as a foundational basis for subsequent quantitative analysis. This analytical approach aims to address the central research question: identifying the core contextualized needs of the older adults in older communities and determining their relative priorities.

The questionnaire focuses on usage demands for community public service facilities. These mainly include four aspects: basic information, behavioral preferences, facility usage experience, and demands and suggestions. In the basic information section, background data, such as gender, age, health status and years of residence, are collected. In the section on behavioral preferences, we ask older adults residents about their daily activity time, the community facilities they often visit, and their activity pace in order to understand their activity characteristics. The facility usage experience section evaluates aspects such as facility usage at different times, the safety of night lighting, and facility multi-functionality using a Likert five-point scale. Finally, in the demands and suggestions section, we set up open-ended questions to solicit opinions on improving the existing community facilities and types of facilities respondents hope to see.

### 3.4 Reliability and validity analysis

In this study, Cronbach’s *α* coefficient was used to test the reliability of the questionnaire’s 7 Likert-scale items. The calculation showed that the reliability coefficient value of the research data was higher than 0.7, indicating that the questionnaire had good internal consistency and could be used for further analysis.

The content validity of the questionnaire items was evaluated using the content validity index. Five experts with backgrounds related to community aging services or environmental design were invited to score the seven items in the questionnaire on a 4-point scale. The results showed that the I-CVI of six out of the seven items was 0.80 or above, and only item Q4 (“There are many ‘non-functional open spaces’ in the community that are actually used as activity venues”) had an I-CVI of 0.60, which is slightly lower than the general standard (I-CVI ≥ 0.78). However, experts believe that this entry has a certain degree of practical representativeness and suggest that it be retained for research purposes. The average content validity index (scale-level CVI, S-CVI/Ave) of the overall scale was 0.89, indicating that the questionnaire has, on the whole, good content validity and can be used as a measurement tool for subsequent surveys and research with older adults residents.

### 3.5 Research results

A total of 25 questionnaires were distributed, and the recovery rate of valid responses was 100%. Through organizing and summarizing the questionnaire results, analysis was conducted on behavioral preferences, facility experience and demand scoring; this was then combined with feedback from the open-ended questions.

#### 3.5.1 Analysis of behavioral preferences

According to statistics on older adults residents’ outings, approximately 80% of older adults residents choose to engage in community activities during the time periods from 6:00 to 9:00 in the morning and from 14:00 to 17:00 in the afternoon, demonstrating a clear tendency toward “avoiding high temperatures” and “off-peak” behaviors. The frequency of activities from 18:00 to 20:00 in the evening is relatively low, reflecting current deficiencies in facilities such as night lighting and safety.

The square and the fitness equipment area were the most frequently chosen areas for activity, at 86.7% and 63.3%, respectively. However, community gardens, corridors and open spaces in front of buildings also have a relatively high utilization rate, indicating that informal spaces are often endowed with practical usage functions, presenting evidence of the “passive activation of non-functional areas.”

In terms of social behavior, approximately 47% of residents indicated “occasional companionship” and 33% chose “frequent” companionship activities; this suggests that, although the need for community interaction is not strong, there is potential for improvement. The selection rate of “relatively slow activity pace” was 73%, indicating that a slower pace and low-intensity participation are the main behavioral tendencies.

#### 3.5.2 Analysis of facility experience and demand tendencies

Table 2 shows a summary of the average scores of the Likert-scale items (with a full score of 5 points). The score data show that, in light of climate change, older adults people have the most urgent need for facilities such as shading and wind protection. Residents also acknowledge the current passive use of non-functional spaces. However, such use implies the potential for more reasonable planning and design for these spaces. In addition, a lack of social spaces and insufficient security guarantees at night are common concerns. Overall, older adults people prefer slow-paced, low-intensity, and light-flow spatial experiences. They tend to use facilities that reflect their sense of participation and value.

**Table 2:** A summary of the average scores of the Likert-scale items.

Project	Average score
Community public facilities are often crowded with people during the morning and evening rush hours	4.3
Insufficient lighting when traveling at night poses safety hazards	4.0
There is a lack of suitable spaces for older adults people to rest quietly or have quiet chats in the community	4.1
There are many “non-functional open spaces” within the community that are actually used as activity venues	4.5
It is hoped that activity facilities can provide shade and shelter from the wind and cope with weather changes	4.7
I prefer quiet, companion-style socializing over lively gatherings	4.2
It is hoped that community facilities can reflect their own value or a sense of “participation”	4.0

#### 3.5.3 Summary of subjective suggestions

In the open-ended questions section, respondents put forward suggestions for improving community public service facilities based on their own daily experiences. Many older adults people reported that the current rest areas and fitness zones lack shade and shelter from the wind, which is very inconvenient when the weather is bad. They hope to add covered seats and fitness equipment to facilitate activities.

Many residents specifically mentioned that the lights in the community are too dim at night, posing a safety hazard. It is suggested that street lamp brightness be increased to help people feel more at ease when going out at night. Some residents have also noticed that there are many unused spaces in the community, such as those in front of buildings and at the corners of corridors. Although these areas do not have specific purposes, they are usually used by people. Respondents proposed simply renovating these places into small spaces that would be suitable for them to chat and rest.

Some community workers added that more residents could be recruited for facility maintenance work, such as via volunteering or by jointly formulating usage rules. In this way, older adults residents can both contribute to improvements and gain a stronger sense of belonging. From these suggestions, it can be seen that what the residents care about most is facilities that are suitable for use, as well as ensuring safety, facilitating communication, and feeling a sense of their own value. These opinions are very helpful for subsequent community renovations.

### 3.6 Supplementary verification

Although the AHP method has advantages such as clear structure and simple operation, there are still certain risks of subjectivity and consistency in the expert scoring process. To enhance the scientificity and consistency control of the multi-index weight determination process, this study, based on the original AHP analysis method, The Measuring Attractiveness by a Categorical Based Evaluation Technique (MACBETH) semantic judgment method was introduced as a supplementary verification tool. This method constructs a consistency judgment matrix through the language description of the importance differences among indicators by experts (such as “moderate difference,” “maximal difference,” etc.), and estimates the weights of each indicator using linear programming. The format of the questionnaire is shown in the Appendix.

## 4 Demand and scenario analyses of community public service facilities

Based on the above research results and literature review, we analyze the demands of the older adults residents in older communities regarding public service facilities:

### 4.1 Demand analysis in the context of time

The activity demands of older adults residents are affected during different time periods and seasons and under different climatic conditions. Their activity patterns throughout the day thus have significant temporal characteristics. The following are the specific demand points with regards to time:

#### 4.1.1 Summary of subjective suggestions

There are obvious differences in the expressed activity needs in the daytime and at night. During the day, older adults residents tend to engage in outdoor activities such as light exercise and social activities, while at night they take walks and relax. However, public facilities in the community lack night lighting and safety measures, which leads to higher safety risks for residents when they are active in the evening or at night.

#### 4.1.2 Activity peaks and facility pressure

The older adults are more active in the early morning and evening. These periods are usually peak times for carrying out morning exercises, taking walks or socializing. However, public facilities are under great pressure during peak hours, resulting in overcrowding and insufficient resources. Therefore, it is necessary to fully consider usage demands during peak hours, rationally allocate resources and provide sufficient support.

### 4.2 Demand analysis regarding spatial context

Spatial elements are reflected in activity space layouts, facility distribution and environmental quality. Residents’ activities in the community are often restricted by spatial layout and passage conditions. These spatial differences have not been fully considered, resulting in many obstacles when using public facilities. The following are the specific demand points in this regard:

#### 4.2.1 There is no requirement for functional space

Public service facilities in existing communities are often functionally oriented, emphasizing clear use, such as for sports and fitness, rest or entertainment, thus neglecting residents’ need for “aimless staying and wandering.” According to actual research, many older adults people like to “walk around casually” in their community. This behavior is not for achieving any specific purpose, but rather out of the psychological need to pass the time, relieve loneliness or seek occasional social encounters. Different states lead to different spatial demands regarding public service facilities. Common community problems, such as fragmented public spaces, monotonous paths, and scarce resting points, all result in a lack of comfortable, safe and attractive spaces for older adults people during their daily walks or wandering.

#### 4.2.2 “Weak interaction” scenario

Single-function and cramped spaces are common existing problems in older communities. Many older adults people are not good at participating in high-intensity and frequently interactive social activities for a long time; nevertheless, they long to avoid loneliness and hope to obtain emotional comfort and a sense of belonging. Most community spaces emphasize communication, interaction and functional use, leading to a lack of quiet, shared spaces designed for such “weak interaction” needs.

### 4.3 Demand analysis regarding behavior

Behavioral elements reflect the diverse behavioral patterns of older adults people in the community and their changing demands for public facilities. These patterns show significant differences due to their different purposes and environments. The following are the specific demand points with regards to behavioral elements:

#### 4.3.1 “Weak interaction” scenario

Community public service facilities lack a consideration of the physiological changes experienced by older adults people, especially in the morning or during cold weather. Due to the decline in their physical functions, such residents exhibit “slow-paced” behavioral characteristics. That is, they usually do not immediately engage in intense exercise or social activities. Instead, they require a process of slowly waking up their bodies and adapting to the environment; they do this by, for example, standing, sitting still, walking slowly, or even just quietly “daydreaming.” However, existing community spaces lack the “buffer zone” required for such behaviors, resulting in older adults residents having nowhere to stay or wait during adaptive activities.

#### 4.3.2 Dynamic wind protection and sun shading

The older adults are particularly sensitive to climate change, especially changes in wind, sunlight and temperature, which often require them to adjust their positions in the external environment to adapt. On days with intense sunlight, there is a frequent need to change seats or get up to adjust positions and look for shade. In autumn and winter, this becomes a need to move to a warmer place. This frequent behavioral adjustment is necessary for avoiding the wind, mitigating sun exposure or keeping warm. Existing older communities lack flexibility and a more humanized design and are thus unable to meet the needs of their older adults residents under dynamic climatic conditions.

#### 4.3.3 “Self-efficacy” as maintenance behavior in older adults people

As physical functions gradually decline, older adults people have a stronger need for “self-efficacy.” Many do not merely wish to be cared for, but long to continue contributing value in community life, particularly by gaining recognition in daily activities and social interactions. More specifically, they hope to showcase their knowledge, experience and skills by “teaching” or “guiding” others. However, community public service facilities often make such residents feel more passive or unable to contribute.

Public service facilities in older communities demonstrate shortcomings in meeting the diverse needs of their residents. The older adults people’s needs present complex characteristics with multiple dimensions and levels, involving aspects such as physical safety, psychological comfort, social interaction and self-actualization. To meet these demands, it is necessary to clarify their relative importance using the analytic hierarchy process to conduct weight analysis; in this way, different demand type priorities can be clarified, providing a scientific basis for optimizing aging-friendly facilities.

## 5 Research results

### 5.1 Constructing a hierarchical structure model for evaluation indicators

**Figure 3:**
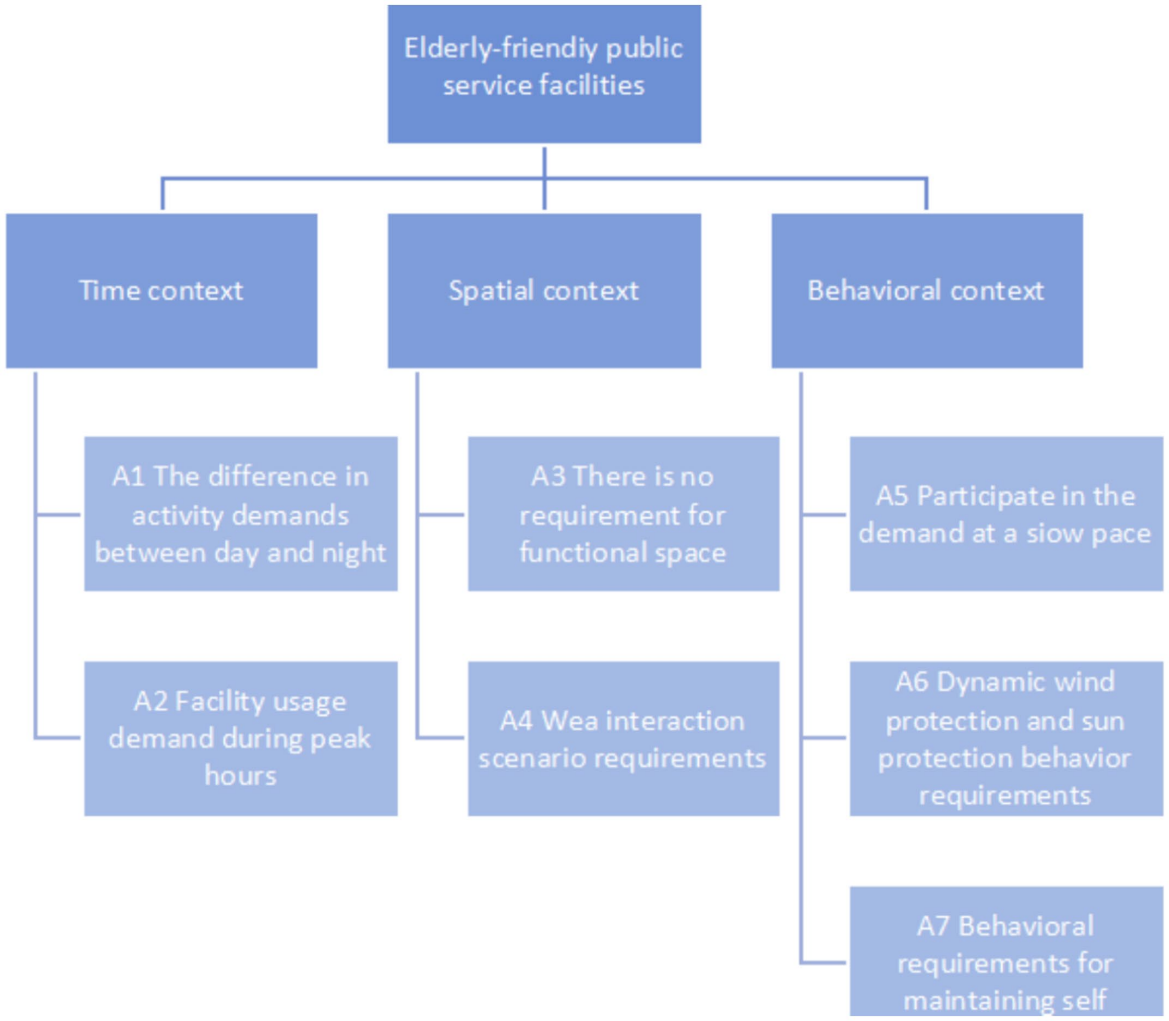
Evaluation index system.

After conducting research on community public service facilities and collating the relevant literature, the relevant elements were summarized. A hierarchical analysis model of public service facilities in aging-friendly communities was constructed, and AHP was used to calculate the index weights in order to provide accurate parameter references. In this case, the evaluation indicators were summarized and screened into three criterion layers and nine sub-criterion layers. Ultimately, an evaluation index system for aging-friendly community public service facilities was constructed, as shown in Figure 3.

### 5.2 Calculating influencing factor evaluation results

We constructed an index judgment matrix based on the above demand evaluation system. Through pairwise comparisons of the evaluation index elements at each criterion layer, corresponding values are assigned according to design element importance, as shown in Table 3.

**Table 3:** Evaluation scale and explanation.

Scale	Meaning
1	The two factors are of the same importance
3	Element a is slightly more important than element b
5	Element a is significantly more important than element b
7	Element a is strongly more important than element b
9	Element a is extremely important compared to element b
2, 4, 6, 8	The median of the above two adjacent judgments
Countdown	If a is relatively important compared to b, mark 5 in row a and column b; when analyzing the importance of b in relation to a, fill in 1/5

**Figure 4:**
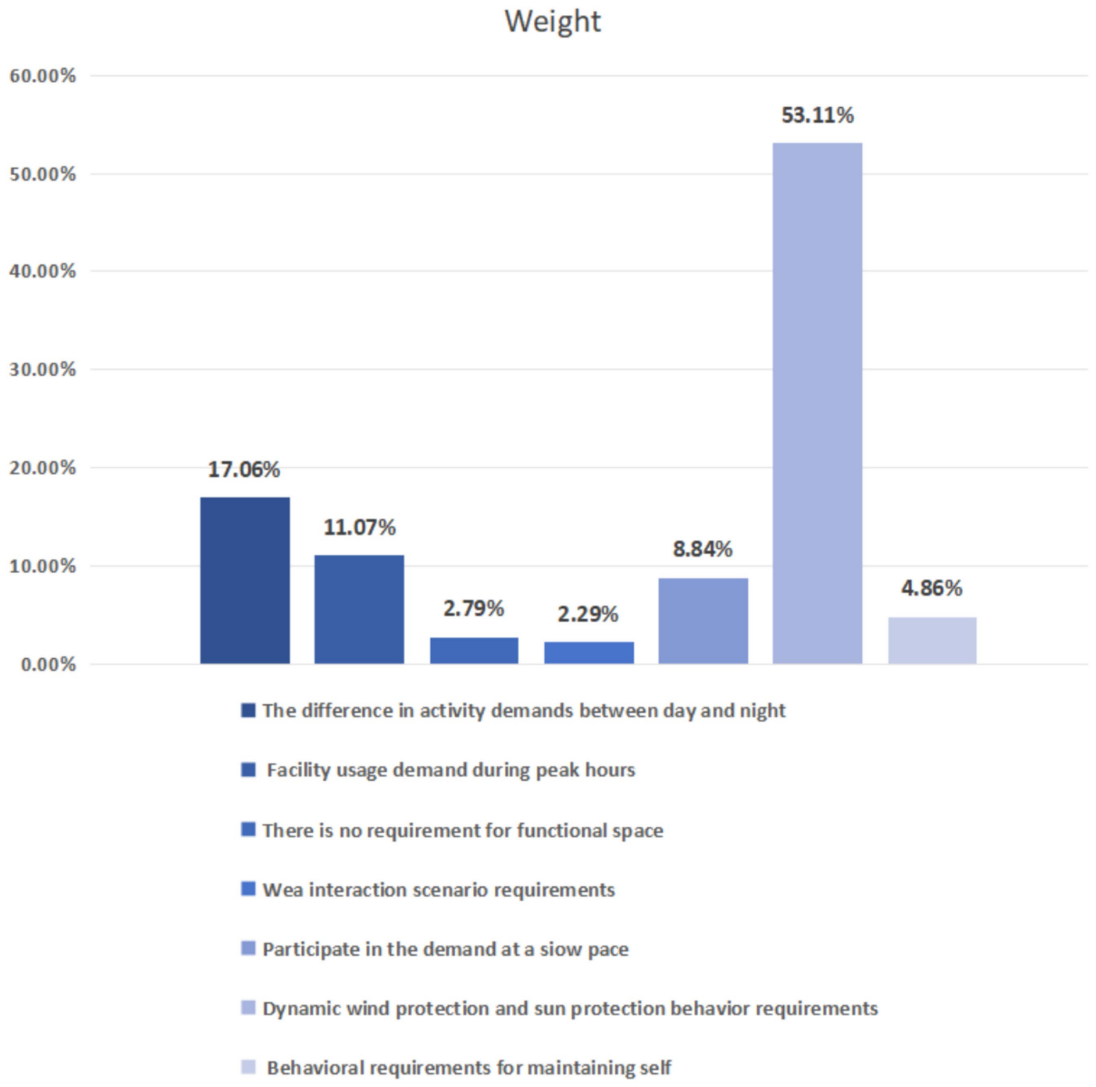
Weight calculation result.

Based on the proposals of four experts—two on-campus master’s supervisors in this field and two professional designers—the indicators of the seven criterion layers were compared. Pairwise comparisons were drawn using the 1–9 scale method before a judgment matrix was established. Combined with the eigenvectors, a maximum eigenroot of 7.757 can be calculated. The CI value calculated for the seventh-order judgment matrix is 0.126, while the RI value in the table is 1.360. Therefore, the calculated CR value is 0.093 < 0.1, which means that the judgment matrix in this study satisfies the consistency test and the calculated weights are consistent. The calculation results are shown in Figure 4.

### 5.3 Consistency test

By analyzing the semantic judgment data of five experts, the results show that “dynamic wind shelter and sunshade behavior requirements,” “differences in circadian activity requirements” and “facility usage requirements during peak hours” have a high consensus of importance among the expert group, reflecting the high dependence of the older adults population on climate adaptability, rhythm responsiveness and spatial organization efficiency in their daily activities.

By conducting semantic judgment and weight calculation on some key evaluation indicators using the MACBETH method, cross-validation was carried out on the results obtained from the original AHP. Analysis shows that the two methods have good consistency in the ranking of main indicators. Meanwhile, the MACBETH method further enhances the consistency of expert judgment and improves the interpretability and stability of the results.

### 5.4 Research limitations and prospects

Current research on community adaptation for the older adults is gradually introducing more dynamic and multi-dimensional data collection and analysis technologies. For instance, GIS-based spatial accessibility modeling, Internet of Things devices for tracking the behavioral paths of the older adults, or digital ethnographic methods for capturing users’ micro-experiences and environmental interactions. As this research is an exploratory practice, we will actively attempt to integrate emerging technological means at the data collection level in the future to further enhance the real-time nature and practical guidance of the research.

## 6 Discussion

### 6.1 Analysis of research results

According to the calculation results, among the seven key factors influencing the aging-friendly design demands of public facilities in older communities, “dynamic wind shelter and shading behavior demands” has the highest weight value. This indicates that environmental regulation dominates the public space usage demands of older adults people, reflecting their high sensitivity in terms of the comfort and safety of outdoor spaces. Under dynamic environmental conditions such as climate change and sunlight intensity, facilities with wind protection and shading functions have a particularly significant impact on ensuring the continuity and health of older adults people’s activities. Therefore, dynamic environmental regulation has become the primary design direction in optimizing aging-friendly facilities. Secondly, the “difference in circadian activity demands” of older adults residents indicates that changes in behavioral patterns over time have a significant impact on facility usage. Differences in activity demands at different times show that public facilities need to be highly adaptable—in terms of usage periods, functional zoning, and lighting safety—in order to meet the differentiated needs of the older adults at various times and in various scenarios, e.g., morning exercise, midday rest, and night-time socializing. “Facility Usage Demand during Peak Hours” emphasizes the concentrated use of public facilities during specific periods. During peak human flow hours, such as early morning and evening, older adults residents’ facility usage frequency increases particularly significantly, leading to higher requirements for aspects such as space capacity, the number of facilities and maintenance. Therefore, in optimizing public spaces in older communities, it is necessary to focus on population distribution characteristics and usage load during peak hours, rationally allocating resources to alleviate the problems of congestion and insufficient resources.

### 6.2 Reasons for the mismatch between community public facility supply and demand

The mismatch between community public facility supply and demand is not accidental, but caused by the combined effects of multiple deep-seated reasons:

#### 6.2.1 The contradiction between fixed facilities and dynamic demands

When designing community public facilities, a fixed pattern is often followed, which neglects to account for the dynamic changes in the needs of older adults residents. In accordance with their diverse lifestyles, their demands for public facilities also show a high degree of contextualization and personalization. However, most public facilities in older communities are still confined to traditional fixed models and lack flexible, adaptive design, resulting in the inability to promptly respond to changes in the needs of their older adults residents.

#### 6.2.2 Facility design lacking multi-scenario adaptation

Existing aging-friendly public facility designs mostly focus on resolving a single, physical barrier, ignoring the complex demands brought about by situational interaction. Many community facilities fail to take into account the changing needs of older adults people in different situations, such as the impact of factors like time, space and behavior on use patterns. A lack of multi-scenario adaptation during construction means that facilities are unable to exert their maximum efficiency in changing environments.

#### 6.2.3 The structural imbalance in facility resource allocation

There is a structural imbalance in the allocation of community public facility resources. Even against the backdrop of an increasingly aging population, the needs of older adults people are often ignored or cannot be met in a timely manner. In the community, the allocation of activity spaces, social spaces and medical facilities for the older adults is insufficient, while other functional facilities are overly concentrated or resources unevenly distributed, further exacerbating the mismatch between facilities and needs.

There are obvious misalignments in the supply and demand structure, as well as in the design concept and usage scenarios for community public facilities. This not only restricts improvements in quality of life for older adults residents, but also affects the overall functional improvement and vitality of the community. In order to solve the problem that existing facilities struggle to meet the diverse and dynamic needs of older adults residents, it is necessary to achieve precise facility–needs matching through multi-dimensional design paths such as dynamic adaptation, context integration, intergenerational integration and data-driven approaches. In this way, balanced and sustainable community service development can be promoted.

### 6.3 Design strategies for aging-friendly public facilities in older communities

In aging-friendly design, common problems include limited resources and tight spaces. Pursuing “all-round” functional coverage is not recommended; instead, “point-to-surface” optimization should be achieved at key demand points. Via analysis of the demands of older adults residents, combined with the problem of supply–demand mismatch, and based on the weight rankings of behavioral demands using hierarchical analysis, a targeted design framework is proposed, aiming to balance the relationship between demands and resources. To this end, situational elements are divided into internal and external factors, which are behavior and the two dimensions of time and space. Based on this, strategies are proposed that strive to achieve efficient response and composite space utilization in a limited space.

#### 6.3.1 The internal factor of behavior: dynamic adaptation facility design

Behavior is the root cause of demand generation, especially in older communities. “Dynamic wind shelter and sun protection,” as the highest-weight behavioral demand, reflects the high sensitivity of older adults people to changes in the microclimate. Therefore, facility design should respond quickly to behavioral changes. The specific strategy is to set up flexible components with shade and wind shelter functions.

Retractable sunshades are flexible and highly adaptable shading systems that are commonly found in semi-open spaces such as balconies and outdoor coffee shops. They can also be transformed into key facilities for aging-friendly shading strategies in older communities. Due to its light weight, retractable sunshade fabric can be flexibly installed in activity areas and quickly unfolded and retracted through mechanical or electric adjustment. Such a system adopts intelligent temperature and light sensing control, automatically forming a shaded area when the temperature is high and retracting when light diminishes. Combined with its soft tones such as off-white and light green, the use of such material not only ensures comfortable use but also maintains environmental harmony. One notable advantage is the preservation of original spatial structures, aligning with the efficient strategy of “unchanged space and variable environment.”

Smart windproof curtains are mainly deployed in communities. They are made of high-strength mesh fabric and are automatically regulated through wind speed sensors. When the wind force exceeds the threshold, it will automatically drop to block the wind; when the wind force weakens, it will automatically retract, all the while retaining the capability of manual adjustment. This kind of intelligent, responsive facility—in which “the environment moves before people do”—reflects a spatial optimization informed by the behavioral needs of older adults residents. More specifically, it represents a direct solution for the high-weight need for “dynamic shelter” in community public spaces.

#### 6.3.2 The external factor of time: scenario-based time period adjustment design

According to our research results, the “difference in circadian activity demands” is also key. The older adults people show different rhythms and behavioral preferences over time. Therefore, public facility usage patterns should also have temporal adaptability. As a non-invasive adjustment method that requires little space, lighting design can effectively guide and support the transformation of behavioral patterns at different times. Particularly in older communities, where space is limited and facility renewal restricted, the rational application of different color temperatures and brightnesses can provide public spaces that are more informed by time and more guided by behavior. Such adaptations are conducive to achieving rhythm control and guaranteeing safety in daily activities.

Early in the day, a neutral color temperature can be adopted to simulate natural morning light, which not only promotes melatonin metabolism but also ensures safety. In combination with sensor floor lamps, such adaptations can guide routes. At night, warm and low-brightness lighting is used. Hidden foot lamps are incorporated into the path, which not only ensures basic visibility but also avoids strong light stimulation. Adding dimmable lamps to rest areas would allow older adults residents to adjust the brightness according to their needs, creating a warm atmosphere. Through scientific light environment design, this sequential lighting strategy effectively enhances comfort and safety during outdoor activities for older adults people at different times of the day and night, without large-scale renovation.

#### 6.3.3 The external factor of space: multi-dimensional chimeric spatial strategies

In older communities, spatial resources are extremely limited. Therefore, space utilization must be maximized through refined and complex strategies. Addressing the problem of overlapping facility usage during peak hours, this study proposes a micro-transformation strategy adopting both a “behavioral overlap area” and “scene transformation area,” emphasizing the efficient reuse of space—through detailed adjustment and light intervention—without making large-scale structural changes.

Take the fitness square as an example. In the morning, it is used as a fitness area, while in the afternoon, it is transformed into a leisure and social area via the addition of folding seats. At night, it is transformed into a walking area using smart light strips. The entire transformation process relies on the original infrastructure, while adapting through local “soft” enhancements. In this way, the complex behavioral needs of older adults residents can be met—fitness, socializing and relaxation at different time periods—and the goals of “behavioral overlap” and “functional elasticity” in a limited space can be truly achieved.

#### 6.3.4 Data-driven facility optimization and iteration

In order to ensure the design continuously adapts to dynamic changes in the behaviors and preferences of older adults residents, it is necessary to conduct long-term tracking and analyze situational changes with the help of a data-driven mechanism. In promoting the continuous, aging-friendly optimization of facilities in older communities, it is necessary to avoid excessive reliance on intelligent systems and instead adopt lightweight and actionable data-driven strategies. For this purpose, basic infrared counters can be deployed in key activity areas to record the flow of people and how long they stay at different times, assisting in determining the peak usage period and the distribution of cold zones. This approach is not only low-cost and simple to operate and maintain, but also avoids disruption for older adults residents.

On this basis, we suggest adopting the pilot mechanism to conduct small-scale experimental optimization in low-efficiency areas with clear data orientation, observing changes in usage before and after the transformation, and then deciding whether to expand its scope. This fine-tuning and progressive feedback mechanism enables facilities to be gradually improved while in use, forming an evolutionary community space that truly meets needs. Through data-driven means, facilities are not only “built” but also “used,” forming an aging-friendly infrastructure that continuously evolves.

## 7 Conclusion

An analysis of different situations provides a dynamic response framework for aging-friendly design, which can adjust according to different elements—such as time, space and behavior—to better meet the constantly changing needs of older adults residents. This theory emphasizes demand dynamics, helping designers to optimize facility configuration and reduce supply–demand mismatch. However, in older communities in China, aging-friendly design faces many challenges, including problems such as aging infrastructures, limited resources, and difficulties in technological adaptation. Applying situational interaction design in such communities is still quite challenging. However, with the development of intelligent technologies and policy support, gradual implementation is expected. The limitations of this study lie in the limited number of cases and the narrow scope of scenario simulation. In the future, through more comparative studies of community cases, combined with technologies such as big data and artificial intelligence, aging-friendly design—both in theory and practice—can be further improved to provide more precise solutions for optimizing community facilities.

## Data Availability

The original contributions presented in the study are included in the article/Supplementary material, further inquiries can be directed to the corresponding author.
