# Supplementary material for: Aging-friendly design strategies for community public service facilities based on context analysis
Source: Front Public Health. 2025 Nov 12;13:1649904. doi: 10.3389/fpubh.2025.1649904 (PMC12646899; doi:10.3389/fpubh.2025.1649904)
Supplement: Supplementary file 1 [file Table_1.pdf]

Appendices A

1. Basic Information

Q1. What is your role?
☐ Elderly resident (living in the community for more than 15 years)
☐ Community administrator
☐ Neighborhood committee member

Q2. Your age (only for elderly residents): ____ years old

2. Behavioral Preferences (Multiple-choice or rating questions)

Q1. During which time periods do elderly residents usually go out for activities? (Select all that apply)
☐ 6:00–9:00 AM ☐ 9:00–11:30 AM ☐ 2:00–5:00 PM ☐ 6:00–8:00 PM ☐ Other: (____)

Q2. Where do elderly residents most frequently engage in activities within the community? (Select all that apply)
☐ Public square ☐ Fitness equipment area ☐ Community garden ☐ Pocket park
☐ Corridors or open space in front of buildings ☐ Other: (____)

Q3. Do elderly residents usually engage in activities with others?
☐ Yes, often ☐ Occasionally ☐ Mostly alone ☐ Never with others

Q4. Do elderly residents generally have a slower pace of activity?
☐ Yes ☐ No ☐ No significant difference

3. Facility Experience and Needs (Likert Scale)

Please rate the following statements based on your personal experience:
(1 = Strongly disagree, 5 = Strongly agree)

Statement	Your Rating (1–5)
Community facilities are often crowded during peak hours	□
Nighttime lighting is insufficient and poses safety risks	□
The community lacks quiet spaces suitable for rest or soft interaction	□
Many “unused spaces” are informally used for activities	□

Statement	Your Rating (1–5)
You wish facilities could provide sunshade, wind protection, and weather adaptability	□
You prefer quiet, companionable social interactions over lively gatherings	□
You hope community facilities offer ways to express personal value or participation	□

4. Open-Ended Suggestions

Q1. In your opinion, which aspect of the current community facilities most needs improvement?

Q2. What types of facilities would you like to see added to improve elderly residents′ daily life?

Appendices B

MACBETH Expert Pairing Judgment Table

Indicator A	Indicator B	Expert Input
A1. Peak-Time Facility Usage	A2. Nighttime Lighting and Safety	
A1. Peak-Time Facility Usage	A3. Quiet Resting Spaces	
A1. Peak-Time Facility Usage	A4. Functional Use of Vacant Land	
A1. Peak-Time Facility Usage	A5. Sunshade and Wind Shelter Facilities	
A1. Peak-Time Facility Usage	A6. Preference for Quiet Socializing	
A1. Peak-Time Facility Usage	A7. Sense of Participation and Value	
A2. Nighttime Lighting and Safety	A3. Quiet Resting Spaces	
A2. Nighttime Lighting and Safety	A4. Functional Use of Vacant Land	
A2. Nighttime Lighting and Safety	A5. Sunshade and Wind Shelter Facilities	

Indicator A	Indicator B	Expert Input
A2. Nighttime Lighting and Safety	A6. Preference for Quiet Socializing	
A2. Nighttime Lighting and Safety	A7. Sense of Participation and Value	
A3. Quiet Resting Spaces	A4. Functional Use of Vacant Land	
A3. Quiet Resting Spaces	A5. Sunshade and Wind Shelter Facilities	
A3. Quiet Resting Spaces	A6. Preference for Quiet Socializing	
A3. Quiet Resting Spaces	A7. Sense of Participation and Value	
A4. Functional Use of Vacant Land	A5. Sunshade and Wind Shelter Facilities	
A4. Functional Use of Vacant Land	A6. Preference for Quiet Socializing	
A4. Functional Use of Vacant Land	A7. Sense of Participation and Value	
A5. Sunshade and Wind Shelter Facilities	A6. Preference for Quiet Socializing	
A5. Sunshade and Wind Shelter Facilities	A7. Sense of Participation and Value	
A6. Preference for Quiet Socializing	A7. Sense of Participation and Value	
